# Guideline-Based Chinese Herbal Medicine Treatment Plus Standard Care for Severe Coronavirus Disease 2019 (G-CHAMPS): Evidence From China

**DOI:** 10.3389/fmed.2020.00256

**Published:** 2020-05-27

**Authors:** Yong-an Ye, The G-CHAMPS Collaborative Group

**Affiliations:** Dongzhimen Hospital, Beijing University of Chinese Medicine, Beijing, China

**Keywords:** COVID-19, Chinese herbal medicine, randomized controlled trial, pilot study, guideline

## Abstract

**Background:** In January, national guidelines were developed and recommended for use throughout China to fight coronavirus disease 2019 (COVID-19). Chinese herbal medicine (CHM) was also included as part of the treatment plans at various stages of COVID-19.

**Methods:** We conducted a pilot randomized, controlled trial in patients with severe COVID-19 in Wuhan, China. Eligible adult patients were randomly assigned in a 2:1 ratio to receive either CHM plus standard care or standard care alone for 7 days. The primary outcome was the change in the disease severity category of COVID-19 after treatment.

**Results:** Between Jan 31, 2020, and Feb 19, 2020, 42 out of 100 screened patients were included in the trial: 28 in the CHM plus standard care group and 14 in the standard care alone group. Among 42 participants who were randomized (mean [SD] age 60.43 years [12.69 years]), 21 (21/42, 50%) were aged ≥65 years, 35 (35/42, 83%) were women, and 42 (42/42, 100%) had data available for the primary outcome. For the primary outcome, one patient from each group died during treatment; the odds of a shift toward death was lower in the CHM plus group than in the standard care alone group (common OR 0.59, 95% CI 0.148–2.352, *P* = 0.454). Three (two from the CHM plus group and one from the standard care alone group) patients progressed from severe to critical illness. After treatment, mild, moderate, and severe COVID-19 disease accounted for 17.86% (5/28) vs. 14.29% (2/28), 71.43% (20/28) vs. 64.29% (9/28), and 0% (0) vs. 7.14% (1/28) of the patients treated with CHM plus standard care vs. standard care alone.

**Conclusions:** For the first time, the G-CHAMPS trial provided valuable information for the national guideline-based CHM treatment of hospitalized patients with severe COVID-19. The effects of CHM in COVID-19 may be clinically important and warrant further consideration and studies.

**Clinical Trial Registration:** http://www.chictr.org.cn/index.aspx. Uniqueidentifier: ChiCTR2000029418.

## Introduction

Approximately 14–16% patients with Coronavirus disease 2019 (COVID-19) suffer from severe diseases like pneumonia, and 5% become critically ill (1, 2). The mortality rate of COVID-19 among those suffering critical illness was reported to be over 50% (2). At present, effective antiviral treatment for COVID-19 is still lacking. Because of continuous widespread and increasing casualties, researchers are racing to find treatments that may speed recovery and lower mortality in COVID-19. The use of Chinese herbal medicine (CHM), such as the classic formula maxingshigantang, yinqiaosan, dayuanyin, xiaochaihutang, et al., in epidemics has a history of thousands of years in China. For example, the use of herbal medicine in malaria ultimately led to the discovery of Artemisinin, an herbal extract from Artemisia annua used as part of the standard treatment worldwide for P. falciparum malaria (3). The herbal formula maxingshigan–yinqiaosan was found to speed fever resolution similarly to oseltamavir for mild H1N1 infection (4). Although showing no mortality benefits, CHM in combination with conventional care might have facilitated pulmonary infiltrate resolution and improved symptoms and quality of life in patients with severe acute respiratory syndrome in the 2002 SARS epidemic (5).

The National Health Commission and the National Administration of Traditional Chinese Medicine of the People's Republic of China developed clinical guidelines for the management of COVID-19 (NHC-NATCM-China guidelines) (6, 7). In these guidelines, CHM was included as part of the treatment plans for severe COVID-19. These recommendations were developed by the consensus of experts. We thus conducted this pilot randomized clinical trial (RCT) to test the potential effectiveness of the guideline-based CHM treatment for severe COVID-19 in Wuhan, China.

## Methods

### Study Design

This was an open-label, pilot, randomized trial for severe COVID-19. The trial was approved by the ethics committee at Dongzhimen Hospital (No. DZMEC-KY-2020-09). The trial was registered at the Chinese Clinical Trial Registry (ChiCTR2000029418). The trial protocol and protocol amendments are provided in Appendices 1-3.

### Patient Enrollment

Patients were screened for eligibility for the G-CHAMPS trial upon admission. During the ongoing epidemic of COVID-19 in Wuhan, China, patients with a confirmatory diagnosis of COVID-19 were directly admitted or transferred to designated COVID-19 hospitals. By Jan 27, 2020, the Chinese government had designated over 40 hospitals for the treatment of COVID-19 in Wuhan. Hubei Provincial Hospital of Integrated Chinese and Western Medicine is one of the hospitals designated by the government for the treatment of COVID-19. Inclusion criteria comprised: adult patients (≥18 years), positive test result for SARS-CoV-2 on a polymerase-chain-reaction (PCR) assay, respiratory rate (RR) ≥30/min or SaO_2_ ≤ 93% or a PaO_2_/FiO_2_ ratio ≤ 300 mmHg (7), and able to provide informed consent. Patients were excluded if known life expectancy was 48 h or less, on home oxygen at baseline, pregnant or lactating, diagnosed with end-stage diseases, or having used immunosuppressants for 6 months or longer. Eligible patients were provided with information about the trial orally and given the opportunity to ask questions. Patients who were willing to take part in the trial were invited for an interview to gather necessary information, including verbal consent; the audio of the interview was electronically recorded.

### Randomization and Masking

Eligible participants were randomized in a 2:1 ratio to the CHM plus standard care (CHM plus) group or the standard care alone group using a simple random allocation method. Allocation was concealed from laboratory personnel and outcome assessors.

### Procedures

Per NHC-NATCM-China guidelines, all patients received standard care, which included hemodynamic monitoring, laboratory testing, supplementary oxygen, intravenous fluids, and routine pharmaceutical medications and other medical care when deemed appropriate by on-duty physicians. Oral ribavirin/arbidole (not remdesivir) was part of the standard care in China (Appendix 1). Per the NHC-NATCM-China guidelines, patients in the CHM plus group also received CHM within 12 h after randomization (Appendix 1); all interventions were in line with updated NHC-NATCM-China guidelines. The herbal formulas were supplied by Jiangyin Tianjiang Pharmaceutical Co., Ltd. The quality of the herbs was in accordance with the 2015 Chinese Pharmacopeia (8). All herbs were tested for heavy metals, microbial contamination, and residual pesticides to ensure that they met the safety standards in China prior to use. Trained and experienced technicians prepared the decoction from the formulas according to a standardized procedure; each unit of formula yielded 400 mL of decoction, divided into two equal portions. Nurses administered 200 mL of the decoction to patients orally (via feeding tube if needed) twice daily for a total of 7 days in the CHM plus group. Data were retrieved from electronic medical records using the standardized case record forms created by members of the ISARIC (9) (International Severe Acute Respiratory and Emerging Infection Consortium) in collaboration with the World Health Organization.

### Outcomes

The primary outcome was the change in the disease severity category of COVID-19 after treatment. The severity of COVID-19 was assessed based on the Six-Point Clinical Status Scale for COVID-19 (COVID-19 severity scale) (Box 1). The Six-Point Clinical Status Scale for COVID-19 was defined according to NHC-NATCM-China guideline and WHO R&D Blueprint. An independent clinical event adjudication committee (CEAC) performed the final outcome assessment based on the pre-specified criteria. Secondary outcomes included the overall survival through last day of treatment, the proportion of patients without improvement (scored 3–5 on the COVID-19 severity scale), the change in serum procalcitonin level after treatment, and the prevalence of antibiotic use during treatment.

Box 1 **The Six-Point Clinical Status Scale for COVID-19**

**Table:** 

0	Hospital discharge or meets discharge criteria	Discharge criteria are defined as: 1 Normal body temperature for more than 3 days; 2 Significantly improved respiratory symptoms: no oxygen supplementation requirement, stable and normal vital signs for longer than 1 day; 3 Lung imaging shows obvious absorption and resolution of acute infiltrates; 4 Negative results of the nucleic acid test for SARS-CoV-2 two times consecutively, with at least a 1-day interval between tests.
1	Mild	Improving and/or mild clinical symptoms and no pneumonia changes in radiological imaging studies.
2	Moderate	Active symptoms like fever and respiratory tract symptoms and pulmonary infiltrates seen in imaging.
3	Severe	Meeting any of the following: 1 Respiratory distress, RR ≥30 breaths/min; 2 Pulse oximetry (SpO_2_) ≤ 93% on room air at rest state; 3 Arterial partial pressure of oxygen (PaO_2_)/oxygen concentration (FiO_2_) ≤ 300 mmHg
4	Critical illness	Meeting any of the following: 1 Mechanical ventilation; 2 Shock; 3 Other organ failure complications that require intensive care unit care
5	Death	

### Statistical Analysis

Since this is a pilot randomized trial, sample size calculation was not performed. For pharmaceutical interventions, a minimum sample size of 12 per group was usually recommended as a rule of thumb for a pilot study (10). Considering a dropout rate of 10%, we aimed to recruit a total sample size of 42 patients (standard care group, *n* = 14; CHM plus group, *n* = 28).

We compared the severity of COVID-19 with ordinal logistic regression (shift analysis). The proportion of patients without clinical improvement after treatment was assessed using the generalized linear model. Laboratory findings were evaluated using the Wilcoxon rank-sum test. Hodges–Lehmann estimates of location shift and 95% CIs are presented.

All outcomes were assessed in the intention-to-treat population with no imputation for missing data. All statistical analyses were performed using SAS version 9.4 (SAS Institute Inc), with a 2-sided *p* < 0.05 considered significant.

## Results

Forty-two out of 100 screened patients were included in the trial (Appendix Figure 1). The two groups were generally well-balanced at baseline, although older patients and more women were enrolled in the CHM plus group than in the standard care alone group (Table 1). Based on symptom-based syndrome differentiation using CHM principles, the included patients in the CHM plus group were divided into the following two syndromes: Lung Blocked by Epidemic Toxin and Inner Blocking Causing Collapse. Correspondingly, the modified formula of maxinshigan–dayuanyin was used in the former, and the shengfutang formula was used in the later syndrome. Lung Blocked by Epidemic Toxin syndrome was found in 20 patients (20/28, 71.43%) and Inner Blocking Causing Collapse in eight patients (8/28, 28.57%) in the CHM plus group. During the G-CHAMPS trial, supportive measures of standard care were similar in the two groups (Appendix 1).

**Table 1:** Baseline demographic and clinical characteristics of the trial population.

	**CHM plus standard care (*n* = 28)**	**Standard care (*n* = 14)**
**Characteristics**		
Age,-yr	65 (53.5–69)	59 (47–67)
Age ≥65 yr, - no. (%)	16 (57)	5 (36)
Age <65 yr, -no. (%)	12 (43)	9 (64)
Sex, no. (%)		
Men	2 (7)	4 (29)
Women	25 (93)	10 (71)
Current smoker, no. (%)	0	0
Heart rate, per min	89 (70–92.5)	97 (90–105)
Blood pressure, mm Hg		
Systolic pressure, mm Hg	129 (110–140)	115.5 (110–119)
Diastolic pressure, mm Hg	85 (74.5–90)	80.5 (75–90)
Body temperature, °C	37 (36.6–37.1)	36.4 (36.2–37)
Respiratory rate >24 breaths, per min	28 (100)	14 (100)
SaO_2_	89 (86–90.5)	89 (87–90)
Transfer from other hospitals-no. (%)	2 (7.41)	4 (28.57)
Onset of symptoms to hospital admission, days	9 (6.5–11.5)	9.5 (6–14)
Hospital admission to randomization, days	1 (0.5–2)	0.5 (0–1)
**Any Comorbidity-no. (%)**		
Chronic heart disease, including congenital heart disease (except hypertension)	8 (28.57)	3 (21)
Chronic lung disease (except asthma)	2 (7.14)	2 (14)
Asthma	1 (3.57)	0
Mild liver disease	3 (10.71)	2 (14)
Chronic nervous system diseases	2 (7.14)	0
Malignant tumor	0	1 (7.14)
Diabetes without complications	1 (3.57)	3 (21.43)
Hypertension	12 (42.86)	7 (50.00)
Hyperthyroidism	0	1 (7.14)
**Presenting Symptoms and Signs-no. (%)**		
History of fever^*^	27 (96)	9 (75)
Cough	23 (82)	12 (86)
Sputum	10 (36)	4 (29)
Sore throat	1 (4)	0
Rhinorrhea	0	1 (7)
Loss of appetite	25 (89)	12 (86)
Insomnia	20 (71)	10 (71)
Wheezing	5 (18)	1 (7)
Chest pain	2 (7)	1 (7)
Muscle pain	8 (29)	6 (43)
Arthralgia	0	1 (7)
Fatigue	26 (93)	14 (100)
Shortness of breath (dyspnea)	5 (18)	5 (36)
Headache	2 (7)	1 (7)
Vomiting/nausea	6 (21)	1 (7)
Diarrhea	3 (11)	3 (21)
Chest x-ray and CT Findings^**^		
Ground-glass opacity	15 (79)	7 (78)
Local patchy shadowing	0	1 (11)
Bilateral patchy shadowing	4 (21)	1 (11)

*CHM= Chinese herbal medicine. Data are presented as median (IQR) unless otherwise indicated*.

* *Two participants in the standard care group had no baseline record of fever*.

** *Chest x-ray and CT findings (standard of care plus CHM, n = 19; standard care group, n = 9). Transfer here was considered as new admission in this trial*.

**Figure 1:**
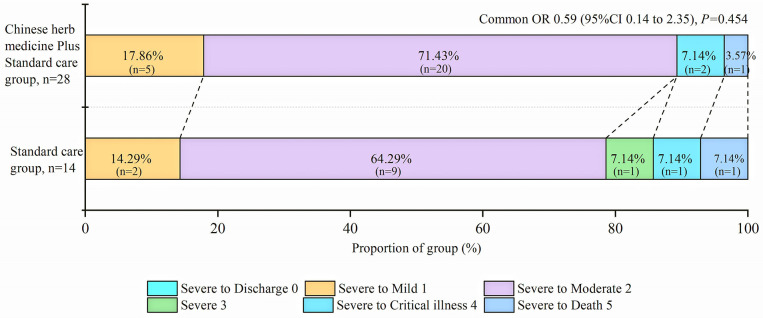
Distribution of COVID-19 severity score at 7 days. OR = odds ratio. The figure denotes scores on the COVID-19 severity scale for patients in the Chinese herbal medicine plus standard care group and the standard care alone group. Scores on the COVID-19 severity scale range from 0 = discharge to 5 = death. A difference between the Chinese herbal medicine plus standard care group and the standard care group was noted in the overall distribution of scores, favoring the Chinese herbal medicine plus standard care group (common odds ratio for improvement of 1 point on the COVID-19 severity scale, 0.59; 95% confidence interval (CI), 0.14–2.35).

For the primary outcome, one patient from each group died during the first 3 days of treatment; the odds of a shift toward death was lower in the CHM plus group than in the standard care group (common OR 0.589, 95% CI 0.148–2.352 *P* = 0.454; Figure 1). The results for the changes shown by imaging studies are listed in Table 2. For secondary outcomes, 11% (3/28) of patients in the CHM plus group and 21% (3/14) of patients in the standard care alone group had no clinical improvement (difference −10.71 (−35.07 to 13.64), *P* = 0.350) after treatment. More secondary outcomes and safety outcomes are provided in Appendix Tables 1–5.

**Table 2:** Imaging features of pneumonia by chest X-ray examination (or chest CT) post-7-day treatment.

**Chest X-ray and CT findings, *n* (%)**	**CHM plus standard care (*n* = 28)**	**Standard care (*n* = 14)**
No pneumonia change	2(8.7)	0
Pneumonia change	21(91.3)	12(100)
Missing data	5	2

## Discussion

To our best knowledge, this is the first prospective randomized trial to investigate the effect of NHC-NATCM-China guideline-based CHM in patients with severe COVID-19. In this trial, the odds of a shift toward death or critical illness at 7 days after treatment was lower in the CHM plus group at a non-significant level. The result was collaborated with the universal normalization or near normalization of leukocytes and different inflammatory markers. In a retrospective study with data of 1,099 patients with COVID-19, 5% (55/1,099) of the patients were admitted to the ICU, 2% (25/1,099) underwent invasive mechanical ventilation, and 1% (15/1,099) died, whereas the composite of these endpoints occurred in 25% of the patients with severe disease (11). In our trial, 12% (5/42) of the patients with severe COVID-19 required ICU care, and 5% (2/42) died within 7 days. That retrospective study collected data from 30 provinces around China, while our trial data are from Wuhan. Disease severity is an important factor when considering treatment for COVID-19 and likely contributed to the differences between these two studies. An ongoing trial of Gilead Sciences' Remdesivir utilized a category ordinal scale to define its primary outcome (NCT04257656).

Although COVID-19 is caused by a virus and will heal without treatment in the majority of patients, most patients in the G-CHAMPS trial received antibiotics. The percentages of antibiotic use are comparable to the previous study (80%) (11).

Animal studies found that the Chinese herbal medicine maxingshigan could decreased lung cell apoptosis and reduced the serum content of TNF-α in acute lung injury from H1N1 infection (12). During the 2002 SARS outbreak, Poon et al. (13) found that herbal medicine had immunomodulating effects in regulating the subgroups of T lymphocytes. Changes in the inflammatory markers seem to aid the hypothesis of a lung-protective effect of CHM in COVID-19. These results of the present trial of CHM in COVID-19 were consistent with previous findings that CHM like maxingshigan can speed up patient recovery in respiratory epidemics (4).

Our study has several limitations, including an open-label design and a small sample size. As with other small studies, a natural manifestation of disease development may influence clinical outcome despite close monitoring. Additionally, this study lacks long-term outcomes, and the COVID-19 disease severity scale deserves further investigation. There is nothing wrong with conducting a well-designed small trial, it just needs to be interpreted carefully. Despite these substantial limitations, the G-CHAMPS trial provided an important opportunity to better understand the use of CHM for severe COVID-19.

For the first time, the G-CHAMPS trial provided valuable information for national guideline-based CHM treatment for hospitalized patients with severe COVID-19. As effective antiviral treatment is still lacking for COVID-19, and SARS-CoV-2 continues to spread outside of China (14), all potentially effective treatments, including CHMs, are worth vigorous further investigation. Adequately powered clinical trials of CHMs are needed to further assess their efficacy and safety for the treatment of severely ill hospitalized COVID-19 patients.

## Data Availability Statement

The data that support the findings of this study are available from the corresponding author on reasonable request. Participant data without names and identifiers will be made available after approval from the corresponding author and the National Health Commission. After publication of the study findings, the data will be available to others on request. The research team will provide an email address for communication once the data are approved to be shared with others. A proposal with detailed description of study objectives and statistical analysis plan will be needed for evaluation of the reasonableness of the request for our data. The corresponding author and the National Health Commission will make a decision based on these materials. Additional materials may also be required during the process.

## Ethics Statement

The studies involving human participants were reviewed and approved by ethics committee of Dongzhimen Hospital. Patients who were willing to take part in the trial were invited for an interview to gather necessary information, including verbal consent; the audio of the interview was electronically recorded.

## Author Contributions

YY, CA, and HS: concept and design. TL, XH, YZ, TW, JD, XG, WH, CJ, DJ, HW, WX, and ZZ: acquisition, analysis, or interpretation of data. CZ and YL: drafting protocol. CZ, YL, KZ, and HS: drafting of the manuscript. CZ, KZ, YL, and HS: critical revision of the manuscript for important intellectual content. YL: statistical analysis. All authors: final approval of the version to be published and dedicated large amounts of time to the study, in the hope of improving care for patients during COVID-19 outbreak. GT, XZ, XW, YC, HD, JZ, and XZ: administrative, technical, or material support. YY: agreement to be accountable for all aspects of the work in ensuring that questions related to the accuracy or integrity of any part of the work are appropriately investigated and resolved. In addition to the core writing group, the group members also contributed substantively to the conduct of the G-CHAMPS trial. These authors contributed equally to this work (Appendix 4 the G-CHAMPS collaborative group). All members: read and approved the final report. All authors agree, as the G-CHAMPS group members, to submit this article.

## Conflict of Interest

The authors declare that the research was conducted in the absence of any commercial or financial relationships that could be construed as a potential conflict of interest.
