# Supplementary material for: Guideline-Based Chinese Herbal Medicine Treatment Plus Standard Care for Severe Coronavirus Disease 2019 (G-CHAMPS): Evidence From China
Source: Front Med (Lausanne). 2020 May 27;7:256. doi: 10.3389/fmed.2020.00256 (PMC7267028; doi:10.3389/fmed.2020.00256)
Supplement: Supplementary file 1 [file Data_Sheet_1.PDF]

Appendix 1. Trial protocol, Pages 2-23;

Appendix 2. Protocol Amendments, Pages 24-25;

Appendix 3. Guidance for Corona Virus Disease 2019: Prevention, Control, Diagnosis and Management, Pages 26-36;

This supplement contains the following items:

Original protocol (version 1.0) andsummary of approved amendments.

PROTOCOL

A pilot randomized controlled trial of guideline based Chinese herbal medicine treatment for hospitalized patients with severe coronavirus disease

Version: 1.0

(January 30, 2020)

Principal Investigator

Yong'an Ye

Dongzhimen Hospital Affiliated to Beijing University of Chinese Medicine

5 Haiyuncang Lane, Dongcheng District

Tel.: 010-84013190; Email: dzmscholar@163.com

TABLE OF CONTENTS

LIST OF ABBREVIATIONS 7

PROTOCOL SUMMARY 8

1.BACKGROUND INFORMATION AND SCIENTIFIC RATIONALE 10

BACKGROUND 10

2.STUDY OBJECTIVES 12

PRIMARY OBJECTIVE 12
SECONDARY OBJECTIVES 12

3.STUDY DESIGN 12

OUTCOMES 12

4.STUDY POPULATION 14

RECRUITMENT 14
INCLUSION CRITERIA 14
EXCLUSION CRITERIA 14
SUBJECT WITHDRAWAL 14
DISCONTINUATION OF SUBJECT BY INVESTIGATOR 14

5.TREATMENT 15

RANDOMISATION AND BLINDING 15
INTERVENTIONS 15

6.STUDY PROCEDURES 17

PERSONNEL FOR STUDY PROCEDURES 17
CASE REPORT FORM 17
SCHEDULE OF ASSESSMENTS 17

7.STATISTICAL METHODS 19

8.ASSESSMENT OF SAFETY 20

DOCUMENTING, RECORDING, AND REPORTING ADVERSE EVENTS 20
DEFINITIONS 20
DATA AND SAFETY MONITORING BOARD (DSMB) 20

9.ETHICAL CONSIDERATIONS 21

10.PUBLICATION PLAN 22

LIST OF ABBREVIATIONS

AE Adverse Event

CDC Center for Disease Control and Prevention

CEAC Clinical Event Adjudication Committee

CI Confidence Interval

ChiCTR Chinese Clinical Trial Registry

CRF Case Report Form

DSMB Data and Safety Monitoring Board

GC Guideline based Chinese medicine

GMP Good Manufacturing Practices

ICF Informed Consent Form

IEC Institutional Ethics Committee

IRB Institutional Review Board

ISARIC International Severe Acute Respiratory and Emerging Infection Consortium

NHC-China National Health Commission of the people’s republic of China

NATCM-China National administration of traditional Chinese medicine of the people’s republic of China

NMPA National Medical Products Administration

PCT Procalcitonin

PI Principal Investigator

RCT randomised controlled trial

RR Respiratory Rate

PCR Polymerase-Chain-Reaction

SAE Serious Adverse Event/Serious Adverse Experience

SOC standard of care

PROTOCOL SUMMARY

**Full Title**<br>A pilot randomised controlled trial of guideline based Chinese herbal medicine treatment for hospitalised patients with severe 2019 novel coronavirus infected pneumonia
**Short Title** The G-CHAMPS TRIAL
**Principal Investigator**<br>Yong'an Ye
**Study Design** Pilot randomized controlled trial
**Accrual Period** January 2020 – March 2020
**Sample Size** 42
**Study Population** Patients with known severe 2019 novel coronavirus infected pneumonia
**Conducted by**<br>Hubei Provincial hospital of Integrated Chinese and Western Medicine, Wuhan, China
**Background**<br>Since December, 2019, a number of novel coronavirus-infected pneumonia patients have been found in Wuhan, Hubei, which has also been found in other parts of China and even abroad as the epidemic spread. The clinical spectrum of 2019 novel coronavirus infected pneumonia ranges from mild to critically ill cases. The mortality of critically ill patients is high. There is a great need for therapeutics for human coronavirus infections that can improve clinical outcomes, speed recovery, and reduce the requirements for ICU and lower mortality. With the in-depth understanding of 2019 novel coronavirus and the accumulation of diagnosis and treatment experience, National Health Commission of the people's republic of China (NHC-China) and National administration of traditional Chinese medicine of the people's republic of China (NATCM-China) has released a clinical guideline for 2019 novel coronavirus. The national guideline covers all hospitals in China and has a huge impact on novel coronavirus care, which Chinese medicine treatment has to be guided by consensus opinion or clinical experience until better evidence is available. So identify clinical evidence as soon as possible is critical for the response to the 2019 novel coronavirus outbreak.
**Objectives**<br>**Primary Objective**: To test potential effectiveness and the feasibility of guideline based Chinese medicine (GC) treatment plus SOC for severe 2019 novel coronavirus-infected pneumonia.<br>**Secondary Objectives:**<br>● To evaluate the comparative effects of investigational therapeutics (GC+SOC vs SOC alone) on clinical parameters of severe 2019 novel coronavirus-infected pneumonia.<br>● Comparative frequency of adverse events (AEs) and serious adverse events (SAEs).
**Interventions**<br>Arm A: GC treatment + SOC<br>Arm B: SOC alone

**Inclusion Criteria**
● Men or women (≥ 18 years) who had positive test results for 2019 novel coronavirus on a polymerase-chain-reaction (PCR) assay and from whom informed consent could be obtained are eligible.<br>● Hospitalised patients of sufficient severity with respiratory rate (RR) ≥ 30/min or SaO2≤93% or a Pa02/Fi02 ratio ≤300mmHg.
**Exclusion Criteria**
● Severe basic diseases that affect survival, including: uncontrolled malignant tumors with multiple metastases that cannot be removed, hematological diseases, cachexia, active bleeding, severe malnutrition, HIV, etc.;<br>● Obstructive pneumonia caused by lung tumors, severe pulmonary interstitial fibrosis, alveolar proteinosis, allergic reaction alveolitis;<br>● Previous immunosuppressive agents within 6 months before study entry or recipients of a solid-organ or bone marrow transplant;<br>● Pregnant or lactating women;<br>● Researchers believe that patients may have other factors affecting the efficacy or safety evaluation of this study.
**Outcomes**
Primary outcome: Critically ill patients (%) after 7 days' treatments.<br>Secondary outcome: PaO_2/FiO_2, serum procalcitonin (PCT), and the prevalence of antibiotic use.

1.BACKGROUND INFORMATION AND SCIENTIFIC RATIONALE

Background

Since December, 2019, a number of new coronavirus-infected pneumonia patients have been found in Wuhan, Hubei, which has also been found in other parts of China and even abroad as the epidemic spread. The disease has been included in the Category B infectious diseases stipulated in the Law of the People's Republic of China on the Prevention and Treatment of Infectious Diseases, and the prevention and control measures for Category A infectious diseases have been taken. As on January 30, there were 9692 confirmed coronavirus cases reported in China (http://www.nhc.gov.cn/xcs/yqtb/202001/a53e6df293cc4ff0b5a16ddf7b6b2b31.shtml).

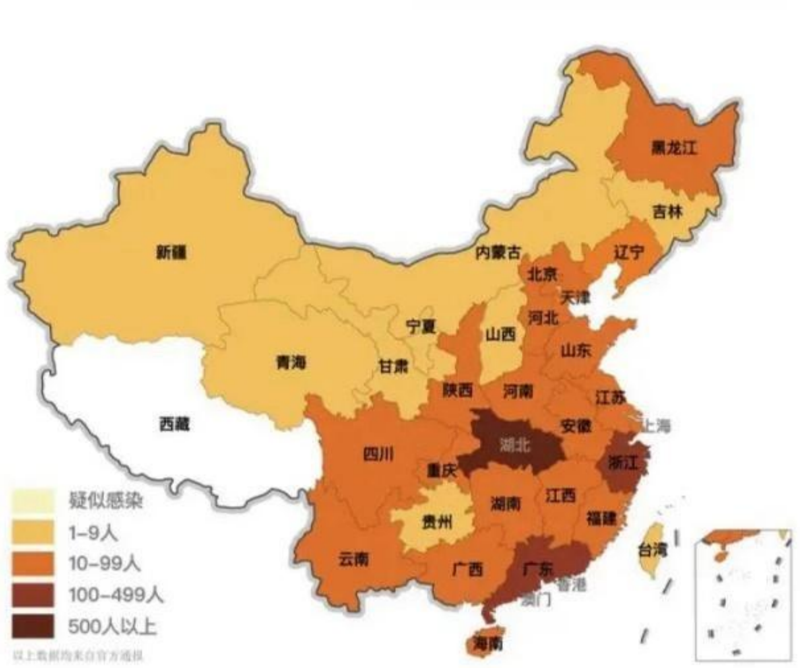

Figure 1 Novel coronavirus infection map (in Chinese, January 30, 2020).

The main manifestations include fever, debilitation, and dry cough. A few patients also have nasal congestion, runny nose, diarrhea and other symptoms. In severe cases, dyspnea usually occurs after one week, and it will rapidly progress to acute respiratory distress syndrome, septic shock, incorrigible metabolic acidosis, and bleeding and coagulation dysfunction[1]. It is worth noting that patients with severe or critical illness may have moderate or low fever, or even no significant fever. People are generally susceptible. Older people and people with chronic diseases are more serious after infection. Deaths are more common in the elderly and those with chronic diseases.

At present, specific antiviral treatment for novel coronavirus infection is still lacking. Because of its wide spread and increasing casualties, researchers are racing to find treatments which may speed recovery and lower mortality in novel coronavirus infection. The use of Chinese medicine in epidemics has a history of thousands of years in China. The herbal decoction maxingshigan–yinqiaosan was found to speed fever

resolution similarly to oseltamavir for mild H1N1 infection[2]. On January 23, 2020, National Health Commission of the people's republic of China (NHC-China) and National administration of traditional Chinese medicine of the people's republic of China (NATCM-China) publicly released a 3rd edition clinical guideline for new coronavirus-infected pneumonia[3]. The third edition first added Chinese medicine treatment. Four days later, updated edition has been issued[4]. The national guidelines were recommended to use by clinicians for the treatment of novel coronavirus infection throughout China. These recommendations were developed by the consensus of experts of traditional Chinese medicine with extensive clinical experiences in both respiratory medicine and Chinese herbal medicine. So identify clinical evidence as soon as possible is critical for the response to the new coronavirus outbreak.

2. STUDY OBJECTIVES

Primary Objective: To test potential effectiveness and the feasibility of guideline based Chinese medicine (GC) treatment plus standard care for severe 2019 novel coronavirus-infected pneumonia.

Secondary Objectives:

- To evaluate the comparative effects of investigational therapeutics on clinical parameters of severe 2019 novel coronavirus-infected pneumonia.
- Comparative frequency of adverse events (AEs) and serious adverse events (SAEs)

3. STUDY DESIGN

A pilot randomised, controlled clinical trial of GC plus standard of care (SOC) compared to SOC. Treatment efficacy evaluations are based on outcome comparisons between treatment arms from concurrently enrolled subjects. CONSORT for Pilot and Feasibility Trials Flow Diagram as follows (Figure 1).

Outcomes

The primary outcome of this trial will be comparative critically ill patients (%) at 7 days' treatments, with a number of secondary outcomes that hopefully will generate generalizable knowledge of GC in severe cases: PaO_2/FiO_2, serum procalcitonin (PCT), and the prevalence of antibiotic use.

Figure 1 CONSORT for Pilot and Feasibility Trials Flow Diagram[5]

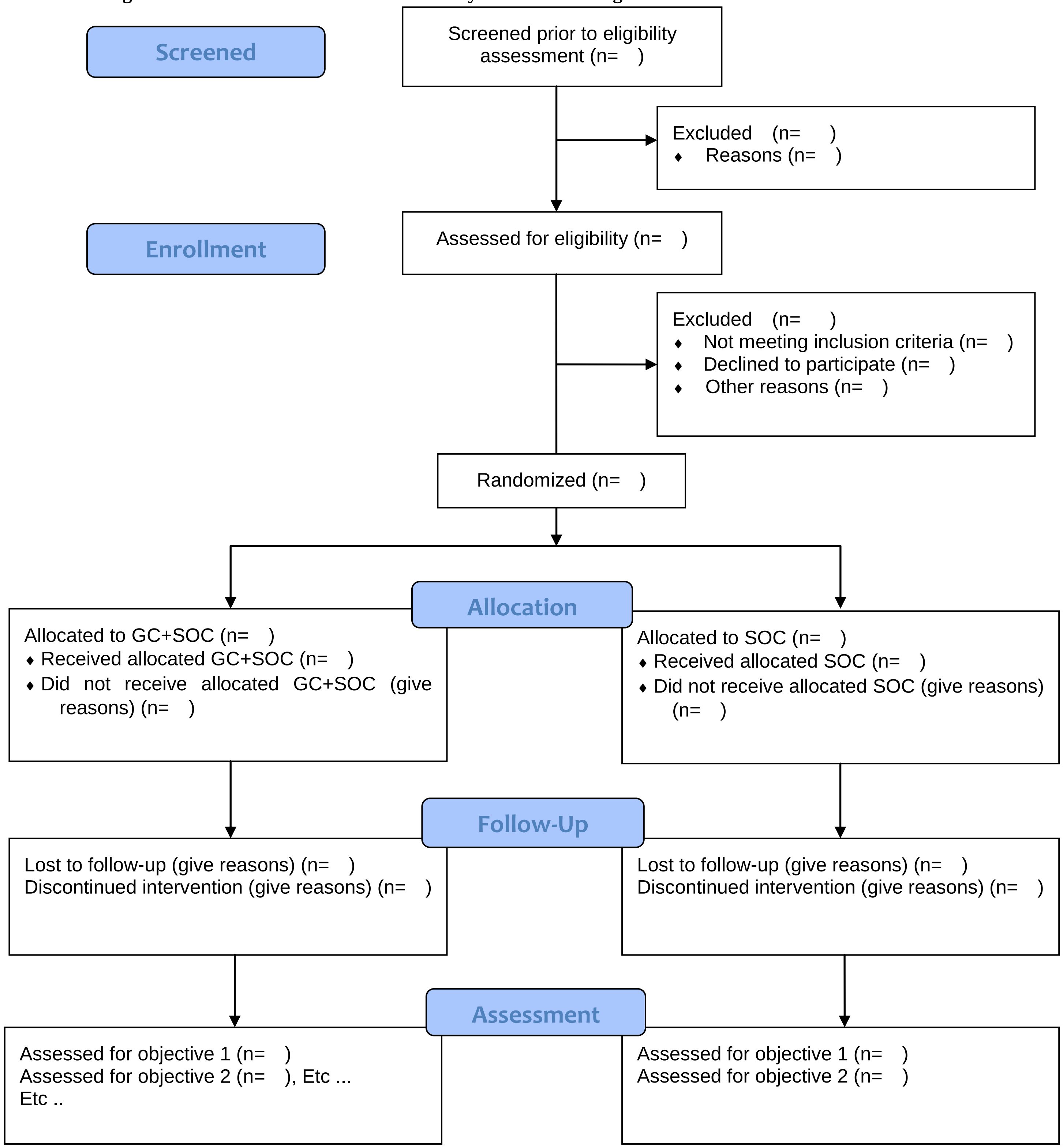

4. STUDY POPULATION

Recruitment

Enrollees will be sought from amongst those individuals who are hospitalised in Hubei Provincial Hospital of Integrated Chinese and Western Medicine.

Inclusion Criteria

- Men or women (≥ 18 years) who had positive test results for 2019 novel coronavirus on a polymerase-chain-reaction (PCR) assay and from whom informed consent could be obtained are eligible.
- Hospitalised patients of sufficient severity with respiratory rate (RR) ≥ 30/min or $SaO_2 \leq 93\%$ or a $Pa0_2/Fi0_2$ ratio ≤300mmHg.

Exclusion Criteria

- Severe basic diseases that affect survival, including: uncontrolled malignant tumors with multiple metastases that cannot be removed, hematological diseases, cachexia, active bleeding, severe malnutrition, HIV, etc.;
- Obstructive pneumonia caused by lung tumors, severe pulmonary interstitial fibrosis, alveolar proteinosis, allergic reaction alveolitis;
- Previous immunosuppressive agents within 6 months before study entry or recipients of a solid-organ or bone marrow transplant;
- Pregnant or lactating women;
- Researchers believe that patients may have other factors affecting the efficacy or safety evaluation of this study

Subject Withdrawal

Subjects can terminate study participation at any time without prejudice. If a subject terminates participation before completing the study, the reason for this decision will be recorded in the study record. Best efforts will be made to follow withdrawn subjects who have received study interventions for safety.

Discontinuation of Subject by Investigator

The investigator has the right to withdraw subjects from the study. Patients may be withdrawn from the study for any of the following reasons:

- The investigator believes that continuation in the study would be detrimental to the subject. Subjects withdrawn for AEs will still be followed for safety follow-up if possible.
- If in the investigator’s best judgment discontinuation is in the subject’s best interest.

The reason for withdrawal from the study is to be recorded in the study record. The investigator is to make every attempt to follow all AEs to resolution.

5. TREATMENT

Randomisation and blinding

This study follows an open-label randomisation design. Given the difficulties of recruitment for the standard care alone group in this trial, an unequal randomisation of 2:1 is chosen in this trial. An independent company without any other involvement of the trial produced the randomization list. The onsite physicians request the randomization number (group allocation) via cellphone software. Patients and onsite investigators who are also involved in patients' care are not masked to treatment allocation. Allocation will be concealed to laboratory personnel and outcome assessors.

Interventions

Per NHC NATCM China guideline, all patients received standard care, which included intravenous fluids, hemodynamic monitoring, laboratory testing, supplementary oxygen, and routine pharmaceutical medications and medical care when deemed appropriate by on-duty physicians.

In the GC plus group, Chinese herbal medicine will be used per the NHC NATCM China guidelines within 12 hours after randomisation. Based on symptom-based syndrome differentiation using traditional Chinese medicine principles, the included patients in the GC plus group will be divided into the following two syndromes:

(1) Lung Blocked by Epidemic Toxin

Clinical manifestations: fever not brought down or alternating fever and chills, cough with less phlegm or yellow phlegm, abdominal distension and constipation; chest distress and anhelation, cough and asthmatic suffocation, short of breath when motion; red tongue with yellow & glossy or yellow & dry coating, rapid and slippery pulses.

For patients presented with Lung Blocked by Epidemic Toxin syndrome, the modified maxingshigan formula will be used; the formula is composed of the following herbs: xingren (stir-baked Semen Armeniacae Amarum), 10 g; shengshigao (raw Gypsum Fibrosum), 30 g; gualou (Trichosanthis Fructus) 30g; shengdahuang (Rhei Radix et Rhizoma) 6g (added at the end of decoction preparation); shengmahuang (raw Herba Ephedrae), 6g; zhimahuang (honey-fried Herba Ephedrae), 6 g; tinglizi (Descurainiae Semen) 10g, taoren (Persicae Semen) 10g; caoguo (Tsaoko Fructus) 6g, binlang (Arecae Semen) 10g; cangzhu (Atractylodis Rhizoma) 10g.

(2) Inner Blocking Causing Collapse

Clinical manifestations: dyspnea, frequently asthma or ventilation needed, accompanied by coma, dysphoria, sweating and cold limbs, dark purple tongue with thick & greasy or dry coating, floating and rootless pulse. For patients presented with Inner Blocking Causing Collapse syndrome, the modified shenfutang formula will be used together with Proprietary Chinese Medicine pomanders. Suhexianwan (3g, twice a day with herbal decoction) or Angongniuhuangwan (one pomander, twice a day with herbal decocation). Modified Shengfutang formula is composed of the following herbs: rensheng (Ginseng Radix et Rhizoma), 15g; Heishunpian (Aconiti Lateralis Radix Praeparata), 10g (cook prior to mixture with other

herbs); shanzhuyu (Evodiae Fructus), 15g. Additionally, herbal formulae extract injections are also recommended as needed in the NHC NATCM China guidelines; however, none will be used in the trial. Quality of the herbs is in accordance with the 2015 Chinese Pharmacopoeia. All herbs will be purchased from the same source of company. As a routine in the hospital and prior to the start of the trial, all herbs were tested for heavy metals, microbial contamination, and residual pesticides to ensure they meet the safety standards in China. Trained and experienced technicians prepare the decoction according to a standardized procedure; each unit of formula yielded 400mL of decoction, divided into two equal portions. Nurses will administer the decoction 200mL to patients orally (via feeding tube if needed) twice daily for a total of seven days in the GC group. Additionally, herbal formulae extract injections are also recommended as needed in the NHC NATCM China guidelines; however, none will be used in the trial.

6. STUDY PROCEDURES

Personnel for Procedures

The physical examination and will be performed by two physicians. Two blinded senior physicians reviewed electronic medical charts of all the cases, independently and in duplicate. And two independent, blinded senior thoracic radiologist with 20 years' experience at the central imaging core interpreted relevant imaging data.

Case report form

Data will be obtained from electronic medical records using the standardised case record forms shared by the International Severe Acute Respiratory and Emerging Infection Consortium (ISARIC). (NOVEL CORONAVIRUS ACUTE RESPIRATORY INFECTION CLINICAL CHARACTERISATION DATA TOOL)

Schedule of Assessments

The day when the subject is enrolled and randomized to their assigned treatment arm is denoted as Study Day 0. The first day after enrollment/randomisation is Study Day 1 and will generally be the day in which an investigational treatment intervention will be initiated. Subsequent days will be numbered chronologically through Day 7 of study (Table 1).

Table 1: Schedule of Assessment

	Screen	Baseline	Treatment						
	**-2 Days**	**D0**	**D1**	**D2**	**D3**	**D4**	**D5**	**D6**	**D7**
Screening procedures	X								
PCR assay test	X								
Basic information[a]		X							
Physical examination with vital signs	X	X							
Symptoms and signs		X	X	X	X	X	X	X	X
Laboratory inspection		X							X
Comorbidity		X							
Severity category of covid-19									X
Blood routine	X	X							X
Arterial blood gas	X								X
Coagulation function		X							X
Blood biochemistry		X							X
Infection-related biomarkers		X							X
Complications		X	X	X	X	X	X	X	X
Chest x-ray or CT	X	X							X
Adverse and serious adverse events[b]			X	X	X	X	X	X	X
Concomitant medications[c]			X	X	X	X	X	X	X

[a] Basic information include patient's demographic information, disease history.
[b] Non-serious and serious adverse events will be reported through days 7.
[c] Concomitant medication will be recorded at baseline and during hospitalisation.

7. STATISTICAL METHODS

Since this is a pilot study, a sample size calculation will be not performed. Julious suggested a minimum sample size of 12 per group rule of thumb for a pilot study[6]. Considering a dropout rate of 10%, we will recruit a total sample size of 42 patients.

Summary tables (descriptive statistics and/or frequency tables) will be provided for all variables as appropriate. For continuous variables, means and standard deviations will be presented, unless the variable has a skewed distribution, in which medians, 25th and 75th percentiles will be presented. For primary analysis, we will be analyzed by using a generalized linear model with a binomial distribution. For other categorical data will be compared between groups using a Wilcoxon rank-sum test, chi-square test or Fisher's exact test, as appropriate. Missing data on any outcome will be not imputed.

For all statistical analyses, SAS 9.4 software will be used. All hypothesis testing will be carried out at the 5% (2-sided) significance level.

8. ASSESSMENT OF SAFETY

Regulatory requirements, including National Medical Products Administration (NMPA) regulations and ICH Guideline for Good Clinical Practice, set forth safety monitoring and reporting responsibilities of Sponsors and Investigators to ensure the safety and protection of human subjects participating in clinical trials.

Documenting, Recording, and Reporting Adverse Events

At each contact with the subject, information regarding adverse events will be elicited by appropriate questioning and examinations and will be immediately documented in the subject's medical record/source document and recorded on electronic database.

Definitions

Adverse Event (AE)

An adverse event is any untoward or unfavorable medical occurrence in a human subject, including any abnormal sign (e.g., abnormal physical exam or laboratory finding), symptom, or disease, temporally associated with the subject's participation in the research, whether or not considered related to the research.

Adverse Reaction (AR)

An adverse event that is caused by an investigational agent (drug or biologic).

Suspected Adverse Reaction (SAR)

An adverse event for which there is a reasonable possibility that the investigational agent caused the adverse event. 'Reasonable possibility' means that there is evidence to suggest a causal relationship between the drug and the adverse event. A suspected adverse reaction implies a lesser degree of certainty about causality than adverse reaction, which implies a high degree of certainty.

Serious Adverse Event (SAE)

A Serious Adverse Event is an AE that results in one or more of the following outcomes: death, a life threatening event, an inpatient hospitalization or prolongation of an existing hospitalization, a persistent or significant incapacity or substantial disruption of the ability to conduct normal life functions, a congenital anomaly/birth defect, or a medically important event.

Data and Safety Monitoring Board (DSMB)

The DSMB will review the study data to evaluate the safety, efficacy, study progress, and conduct of the study. All serious adverse events, all unanticipated problems, and all Safety Reports will be reported by the PI to the DSMB at the same time they are submitted to the IRB or IND Sponsor. The PI will notify the Board at the time pausing or halting criteria are met and obtain a recommendation concerning continuation, modification, or termination of the study. The PI will submit the written DSMB summary reports with recommendations to the IRB.

9. ETHICAL CONSIDERATIONS

Regulatory and Ethical Considerations

The trial will be conducted in accordance with all applicable regulatory requirements. The trial will also be conducted in accordance with ICH-GCP guidelines, all applicable subject privacy requirements, and the guiding principles of the Declaration of Helsinki.

Changes to the Protocol

The clinical trial procedures may be changed, and if the changes are substantial, must approve/acknowledge the changes before they can be implemented. All substantial changes must be documented by protocol amendments, if applicable.

10. PUBLICATION PLAN

The trial will be registered at Chinese Clinical Trial Registry (ChiCTR) http://www.chictr.org.cn/index.aspx. ChiCTR was established is 2005, and assigned to be the representative registry of China to join WHO ICTRP in 2007. The results of the trial, positive as well as negative, will be published by the end of the trial. If the results of the pilot trial are to be published in a journal, the authorship credit should be based on (1) substantial contributions to conception and design, acquisition of data, or analysis and interpretation of data; (2) drafting the article or revising it critically for important intellectual content; (3) final approval of the version to be published. Authors should meet conditions 1, 2, and 3.

References

1. Huang C, Wang Y, Li X, Ren L, Zhao J, Hu Y, et al. Clinical features of patients infected with 2019 novel coronavirus in Wuhan, China. Lancet. 2020; **395**(10223): 497-506.

2. Wang C, Cao B, Liu QQ, Zou ZQ, Liang ZA, Gu L, et al. Oseltamivir compared with the Chinese traditional therapy maxingshigan-yinqiaosan in the treatment of H1N1 influenza: a randomized trial. Annals of internal medicine. 2011; **155**(4): 217-25.

3. (NATCM-China) NHCotPsRoCN-CaNAoTCMotPsRoC. Guidance for Corona Virus Disease 2019: Prevention, Control, Diagnosis and Management (Tentative 3rd edition). 2020.

4. (NATCM-China) NHCotPsRoCN-CaNAoTCMotPsRoC. Guidance for Corona Virus Disease 2019: Prevention, Control, Diagnosis and Management (Tentative 4th edition). 2020.

5. Eldridge SM, Chan CL, Campbell MJ, Bond CM, Hopewell S, Thabane L, et al. CONSORT 2010 statement: extension to randomised pilot and feasibility trials. BMJ. 2016; **355**: i5239.

6. Julious SA. Sample size of 12 per group rule of thumb for a pilot study. Pharmaceutical Statistics. 2005; **4**(4): 287-91.

A pilot randomized controlled trial of guideline based Chinese medicine treatment for hospitalized patients with severe coronavirus disease

Protocol Amendments

Trial Registration: Chinese Clinical Trials Registry ChiCTR2000029418. **Prepared for and approved by the Executive committee of the study:**

Dr. Yong-an Ye, Dongzhimen Hospital, Beijing University of Chinese Medicine, Haiyuncang Lane, Dongcheng District, Beijing 100700, China.

Protocol amendments have been to done to improve the scientific validity of this pilot trial. Given the fact that novel coronavirus was largely unknown to the scientific society and urgent effective treatments for critically ill patients are needed, we designed this pilot randomized controlled trial with the primary outcome 'Critically ill patients (%) after 7 days' treatments'. With the in depth understanding and accumulation of diagnosis and treatment experience of novel coronavirus, and updated guideline issued, the outcomes revision proposed by the core team members approved by the Executive committee of the study. The whole number of outcomes has been increased from 4 to 6.

Table S1: Changes to protocol after start of trial

Protocol	Date	Summary of changes
V2.0	Feb 05, 2020	Change primary outcome into: the change in the disease severity category* of 2019 novel coronavirus disease after 7 days' treatments (day 7 after randomization)
V2.0	Feb 05, 2020	1) Change 'Critically ill patients (%)' into 'Score of 3 to 5 on 2019 novel coronavirus disease clinical status scale at 7 days, n (%)'; 2) Addition of secondary outcomes: Survival at 7 days, n (%); 3) PaO_2/FiO_2 has been removed. 4) Not on home O2 was added as an exclusion criterion
V3.0	Feb 11, 2020	Change '2019 novel coronavirus disease' into COVID-19.

Severity of 2019 novel coronavirus disease in these patients will be assessed based on the Six-Point Clinical Status Scale for 2019 novel coronavirus disease (2019 novel coronavirus disease severity scale): [0] hospital discharge or meet the discharge criteria, [1] mild, [2] moderate, [3] severe, [4] critical illness or [5] death (See Box 1).

Box 1. Six-point clinical status scale for 2019 novel coronavirus disease

0	**Hospital discharge or meet discharge criteria**	Discharge criteria are defined as:<br>1 With normal body temperature for more than 3 days;<br>2 With significantly recovered respiratory symptoms;<br>3 Lung imaging shows obvious absorption and recovery of acute exudative lesion;<br>4 With negative results of the nucleic acid tests of respiratory pathogens for consecutive two times (sampling interval at least 1 day).
1	**Mild**	The clinical symptoms are mild and no pneumonia manifestation can be found in imaging.
2	**Moderate**	Patients have symptoms like fever and respiratory tract symptoms, etc. and pneumonia manifestation can be seen in imaging.
3	**Severe**	Meeting any of the following:<br>1 Respiratory distress, RR $\geq$30 breaths/min;<br>2 Pulse oxygen saturation (SpO_2)$\leq$93% on room air at rest state ;<br>3 Arterial partial pressure of oxygen (PaO_2) / oxygen concentration (FiO_2) $\leq$300 mmHg (1 mmHg=0.133 kPa).
4	**Critical illness**	Meeting any of the following:<br>1 Respiratory failure occurs and mechanical ventilation is required;<br>5.4.2 Shock occurs;<br>3 Complicated with other organ failure that requires monitoring and treatment in ICU.
5	**Death**	

Guidance for
Corona Virus Disease 2019

Prevention, Control, Diagnosis and Management

(Tentative Fourth Edition)

National Health Commission (NHC) of the PRC,

General Office; National Administration of Traditional

Chinese Medicine of the PRC, General Office

January 27, 2020

Since December 2019, an increasing number of cases of novel coronavirus pneumonia (NCP) have been diagnosed in Wuhan, Hubei Province. With the spreading of the epidemic, cases have also been reported in other regions of China and abroad. COVID-19 (officially named as Corona Virus Disease 2019 [COVID-19] by WHO) was urgently classified, by the Law of the People's Republic of China on the Prevention and Treatment of Infectious Diseases as Class B communicable diseases, and is managed as Class A communicable diseases. With the in-depth understanding and accumulation of diagnosis and treatment experience of COVID-19, the Diagnosis and Treatment Plan of Corona Virus Disease 2019 (the Tentative 3rd Revised Edition) was revised and formed the current tentative fourth edition.

1 Pathogenic Characteristics

The novel coronavirus 2019 (2019-nCoV, officially named as severe acute respiratory syndrome coronavirus 2 [SARS-CoV-2] by ICTV) belongs to the genus β, with envelope, round or elliptic and often pleomorphic form, and 60–140 nm in diameter. The virus genetically differs considerably from those of SARSr-CoV and MERSr-CoV. Current studies show that the homology between 2019-nCoV and bat-SARS-like coronavirus (bat-SL-CoVZC45) is over 85%. When cultured in vitro, 2019-nCoV can be found in human respiratory epithelial cells after about 96 hours, while it takes about 6 days to

isolate and culture Vero E6 and Huh-7 cell lines.

Knowledge on the physical and chemical characteristics of coronaviruses is mainly derived from the study of SARSr-CoV and MERSr- CoV. Coronaviruses are sensitive to ultraviolet rays and heat, and can be effectively inactivated by heating at 56 °C for 30 min and lipid solvents such as ether, 75% ethanol, chlorine-containing disinfectant, peroxyacetic acid and chloroform except

chlorhexidine.

2 Epidemiological Characteristics

2.1 Source of Infection

At present, the major source of infection is the patients with COVID-19.

2.2 Route of Transmission

COVID-19 is mainly transmitted by droplets and contact.

2.3 Susceptible Individuals

Humans of all ages are generally susceptible. Old people and people with chronic diseases are more serious after infection, and it also attacks children and infants.

3 Clinical Characteristics

3.1 Clinical Manifestations

Based on current epidemiological investigations, the incubation period is generally 3-7 days, with a maximum of 14 days.

Fever, fatigue and dry coughing are considered the main clinical manifestations, but symptoms such as stuffy nose, runny nose, pharyngalgia, myalgia and diarrhea are relatively less common. In severe cases, dyspnea usually occurs after one week of disease onset, and the worse can rapidly progresses to acute respiratory distress syndrome, septic shock, metabolic acidosis hard to correct, and hemorrhage and coagulation dysfunction, multiple organ failure, etc. It's worth noting that patients with severe or critical illness may have a moderate to low fever, or no fever at all. Some patients only have low fever and slight debilitation, without pneumonia, and recover after 1 week.

From the cases treated currently, most of the patients have a favorable prognosis. Cases with

relatively mild symptoms are common in children. The elderly and people with chronic underlying diseases usually have poor prognosis.

3.2 Laboratory Examination

In the early stage of COVID-19, a normal or decreased total white blood cell count and a decreased lymphocyte count can be found in patients. In addition, increased value of liver enzymes, LDH, muscle enzymes and myoglobin can occur in some patients. In most cases, the laboratory tests show a raised C-reactive protein value and erythrocyte sedimentation rate but a normal procalcitonin value. Among severe patients, D-dimer value is increased and peripheral blood lymphocytes decreased persistently.

The nucleic acid of 2019-nCoV can be detected in biological specimens such as throat swab, sputum, secretions of lower respiratory tract and blood.

3.3 Chest Imaging

In the early stage of COVID-19, the images show that there are multiple small patched shadows and interstitial changes, especially in the lung periphery. As the disease progresses, the images of these patients further develop into multiple ground glass shadows and infiltration shadows in both lungs. In severe cases, lung consolidation may occur. It is seldom to find pleural effusion in patients with COVID-19.

4 Diagnostic Criteria

4.1 Suspected Cases

The suspected cases should be diagnosed through considering both the epidemiological histories and clinical manifestations:

4.1.1 Epidemiology

(1) Having a history of travel or residence in Wuhan or other communities with cases reported within 14 days before the patient's onset; or

(2) Having a contact history with patients with fever or respiratory symptoms from Wuhan or the communities with cases reported within 14 days before the patient's onset; or

(3) Clustering occurrence of cases.

4.1.2 Clinical Manifestations

(1) Fever;

(2) Having the imaging features of pneumonia described above;

(3) In the early stage, a normal or decreased total white blood cell count and a decreased lymphocyte count can be found.

Patients who satisfy any one of the epidemiological exposure histories as well as any two of the clinical manifestations can be diagnosed as suspected cases.

4.2 Confirmed Cases

The suspected cases with one of the following etiological evidences can be diagnosed as confirmed cases:

4.2.1 A positive result of the nucleic acid of 2019-nCoV by real-time fluorescence RT-PCR;

4.2.2 The virus gene sequence is highly homologous to the known 2019-nCoV.

5 Clinical Classifications

5.1 Ordinary Cases

Patients have symptoms like fever and respiratory tract symptoms, etc. and pneumonia manifestation can be seen in imaging.

5.2 Severe Cases

Meeting any of the following:

5.2.1 Respiratory distress, RR ⩾30 breaths/min;

5.2.2 Pulse oxygen saturation (SpO_2)⩽93% on room air at rest state ;

5.2.3 Arterial partial pressure of oxygen (PaO_2) / oxygen concentration (FiO_2) ⩽300 mmHg (1 mmHg=0.133 kPa).

5.3 Critical Cases

Meeting any of the following:

5.4.1 Respiratory failure occurs and mechanical ventilation is required;

5.4.2 Shock occurs;

5.4.3 Complicated with other organ failure that requires monitoring and treatment in ICU.

6 Differential Diagnosis

It is mainly differentiated from influenza virus, parainfluenza virus, adenovirus, respiratory syncytial virus, rhinovirus, human metapneumovirus, SARS coronavirus and other known viral pneumonia, as well as mycoplasma pneumoniae, chlamydia pneumonia and bacterial pneumonia. In addition, it should be differentiated from non-infectious diseases, such as vasculitis, dermatomyositis and organic pneumonia, etc.

7 Case Identification and Report

Medical staff at all levels and types of medical institutions should immediately isolate and treat every suspected case. After in-hospital experts' consultation or attending physicians' consultation, people still be considered as suspected cases need to be reported online within 2 hours. Specimens should be collected and tested for the nucleic acid of 2019-nCoV. Suspected patients should be transferred to the designated hospitals as soon as possible. People intimately

contacted with COVID-19 patients or those even with positive results in common respiratory pathogens test, are also recommended to conduct the pathogenic detection 2019-nCoV in time.

8 Treatment

8.1 Determine the Treatment Place According to the Severity of the Disease

8.1.1 Suspected and confirmed cases should be isolated and treated in designated hospitals with effective isolation and protective conditions. Suspected cases should be treated in single rooms, while confirmed cases can be admitted to the same ward.

8.1.2 Critical cases should be admitted to ICU as soon as possible.

8.2 General Treatment

8.2.1 Rest patients in bed, strengthen supportive treatment, and ensure adequate nutrition. Keep the balance of water and electrolyte to maintain the stability of the internal environment. Closely monitor vital signs, oxygen saturation, etc.

8.2.2 Monitor blood routine, urine routine, CRP, biochemical indicators (liver enzyme, myocardial enzyme, renal function, etc.), coagulation function, arterial blood gas analysis, chest imaging, etc. according to the patient’s condition.

8.2.3 According to the change of oxygen saturation, give effective oxygen therapy measures in time, including nasal cannula, mask oxygen, high-flow nasal oxygen therapy.

8.2.4 Antiviral Treatment: Give alpha-interferon nebulization (5 million units or equivalent per time for adult, add 2 mL of sterile water for injection, aerosol inhalation twice per day); lopinavir/ritonavir (200 mg/50 mg per capsule, 2 capsules each time, twice per day for adults);

8.2.5 Antibacterial Drug Treatment: unselective or inappropriate use of antibiotics should be avoided, especially in combination with broadspectrum antibiotics. Strengthen bacteriological

monitoring and apply antibiotics when there is evidence of secondary bacterial infection.

8.3 Treatment of Severe and Critical Cases

8.3.1 Treatment Principles: On the basis of symptomatic treatment, actively prevent complications, treat accompanying diseases, prevent secondary infections, and provide organ function support in time.

8.3.2 Respiratory Support

If no improvement is made after non-invasive mechanical ventilation for 2 hours, or patients cannot tolerate non-invasive ventilation, and have increased airway secretions, severe cough, or hemodynamic instability, invasive mechanical ventilation shall be provided in a timely manner. Low-tidal-volume LPVS (Lung protective ventilation strategy) is used in invasive mechanical ventilation to reduce ventilator-related lung injury. If necessary, prostrate ventilation, pulmonary retraction or extracorporeal membrane pulmonary oxygenation (ECMO) shall be used.

8.3.3 Circulation Support

On the basis of adequate fluid resuscitation, improve microcirculation, use vasoactive drugs, and perform hemodynamic monitoring when necessary.

8.3.4 Other Treatments

According to the severity of respiratory distress and the progress of chest imaging, glucocorticoids can be used within a short period of time (3–5 days) as appropriate. Dose does not exceed the equivalent of 1–2 mg/ kg/day of methylprednisolone is recommended. Xuebijing Injection can be given intravenously 100 mL/day, twice a day for treatment; microecological preparation can be used to keep the equilibrium for intestinal microecology and prevent

secondary bacterial infection; convalescent plasma therapy can be considered if conditions permit. Anxiety and fear usually occur in many patients, therefore psychological counseling should be strengthened.

8.4 Traditional Chinese Medicine Treatment

COVID-19 can also be treated with traditional Chinese medicine, which considers it caused by epidemic pathogenic factors located in the lungs. Different regions can refer to the following schemes for dialectical treatment according to the disease condition, local climate characteristics, and different physical conditions.

8.4.1 Medical Observation Period

Clinical Manifestation 1: fatigue with gastrointestinal upset Recommended Chinese Medicine: Huoxiangzhengqi Capsule (pill, oral liquid)

Clinical Manifestation 2: fatigue with fever

Recommended Chinese Medicines: Jinhua Qinggan Granules, Lianhua Qingwen Capsules (granules), Shufeng Jiedu Capsules (granules), Fangfeng Tongsheng Pill (granules),

8.4.2 Clinical Treatment Period

1) Cold Dampness Stagnating Lungs

Clinical Manifestations: fever with aversion to cold or no fever, dry cough, dry pharynx, listlessness, chest tightness, distention and fullness in gastral cavity, or vomiting, loose stool; pale or light red tongue with white greasy fur, floating and soft pulses.

Recommended Prescription:

cangzhu (Rhizoma Atractylodis) 15 g, chenpi (Pericarpium Citri Reticulatae) 10 g, houpo (Cortex Magnoliae Officinalis) 10 g, huoxiang (Herba Pogostemonis) 10 g, caoguo (Fructus Tsaoko) 6 g,

shengmahuang(raw Herba Ephedrae) 6 g, qianghuo(Rhizoma et Radix Notopterygii)10 g, shengjiang(Rhizoma Zingiberis Recens) 10 g, binlang (Semen Arecae) 10 g.

2) Lung Blocked by Epidemic Toxin

Clinical Manifestations: fever not brought down or alternating fever and chills, cough with less phlegm or yellow phlegm, abdominal distension and constipation; chest distress and anhelation, cough and asthmatic suffocation, short of breath when motion; red tongue with yellow greasy or dry fur, rapid and slippery pulses.

Recommended Prescription: xingren (Semen Armeniacae Amarum), 10 g; shengshigao (Gypsum Fibrosum), 30 g; gualou (Trichosanthis Fructus) 30g; shengdahuang (raw Radix et Rhizoma Rhei) 6g (decocted later); shengmahuang (Raw Herba Ephedrae), 6g; zhimahuang (honey-fried Herba Ephedrae), 6 g; tinglizi (Semen Lepidii) 10g, taoren (Persicae Semen) 10g; caoguo (Fructus Tsaoko) 6g, binlang (Semen Arecae) 10g; cangzhu (Rhizoma Atractylodis) 10g. Xiyanping Injection or Xuebijing Injection.

3) Internal Block and Outward Desertion

Clinical Manifestations: dyspnea, asthma requires assisted ventilation, dizziness, irritability, cold sweaty limbs, purple tongue, thick or dry fur, large floating and rootless pulse.

Recommended Prescription: rensheng (Radix Ginseng) 15 g, Heishunpian (Radix Aconiti Lateralis Preparata)10 g (decocted first), shanzhuyu (Fructus Corni) 15 g, drinking with Suhexiang Pills or Angong Niuhuang Pills. Xuebijing Injection, Shenfu Injection, or Shengmai Injection.

4) Lung Deficiency and Spleen Qi

Clinical Manifestations: shortness of breath, tiredness, anorexia, distention and fullness, constipation, loose stool, pale tongue, whitish greasy fur.

Recommended Prescription: fabanxia (Rhizoma Pinelliae Preparatum) 9 g, chenpi (Pericarpium Citri Reticulatae) 10 g, dangshen (Radix Codonopsis) 15 g, zhihuangqi (roasted Radix Astragali seu Hedysari) 30 g, Poria 15 g, fuling (Herba Pogostemonis) 10 g, sharen (Fructus Amomi Villosi) 6 g (decocted later).

9 Release of Isolation and Discharge Standards

With normal body temperature for more than 3 days; With significantly recovered respiratory symptoms; With negative results of the nucleic acid tests of respiratory pathogens for consecutive two times (sampling interval at least 1 day).

10 Transfer Principle

Special vehicles should be used for the transport of patients, and personal protection and vehicle disinfection of the transport personnel should be implemented. In accordance with the Novel Coronavirus Pneumonia Case Transfer Program (Tentative Edition) issued by our Commission.

11 Hospital Infection Control

Strictly comply with the requirements of the Technical Guide for the Prevention and Control of Novel Coronavirus Infection in Medical Institutions (First Edition) and Guidelines for Usage of Common Medical Protective Equipment in Protection of Novel Coronavirus Pneumonia (Tentative Edition).
