# Supplementary material for: Guideline-Based Chinese Herbal Medicine Treatment Plus Standard Care for Severe Coronavirus Disease 2019 (G-CHAMPS): Evidence From China
Source: Front Med (Lausanne). 2020 May 27;7:256. doi: 10.3389/fmed.2020.00256 (PMC7267028; doi:10.3389/fmed.2020.00256)
Supplement: Supplementary file 2 [file Data_Sheet_2.PDF]

Supplemental Digital Content – Table 1,2,3,4,5 page2-6

Appendix Table 1: Secondary outcomes in the intention-to-treat population

Appendix Table 2: Laboratory data of 42 patients with server COVID-19 at baseline

Appendix Table 3: The frequency of complications for the GC plus group vs the standard of care alone group using descriptive statistics for the intention-to-treat populations

Appendix Table 4: The frequency of treatments for the GC plus group vs the standard of care alone group using descriptive statistics for the intention-to-treat populations

Appendix Table 5: Laboratory data of 40 patients with server COVID-19 at day 7

Appendix Table 1: Secondary outcomes in the intention-to-treat population

Secondary outcomes	Standard of Care plus GC (n=28)	Standard of Care (n=14)	Difference (95%CI)*	P Value
Survival at 7 days, n (%)#	27 (96)	13 (93)	3.57 (-11.57 to 18.71)	0.6084
Score of 3 to 5 on COVID-19 clinical status scale at 7 days, n (%)†	3 (11)	3 (21)	-10.71 (-35.07 to 13.64)	0.3496
Procalcitonin, ng/mL**	0.02 (0.02-0·05)	0·012 (0.02-0·03)	0 (0 to 0.03)	0.3229
Antibiotic use, n (%)	23 (82)	10 (71)	10·71 (-16.88 to 38.30)	0.4250

GC=guideline based Chinese herbal medicine treatment. Data are expressed median (IQR) unless otherwise indicated.

#Overall survival at 7 days defined as the period from the first day of assigned treatment to 7 days.

†COVID-19 clinical status scale ranges from 0 (discharge) to 5 (death), with higher scores indicating worse prognosis.

*Calculated using a generalized linear model.

**Hodges–Lehmann estimate of location shift (pseudo-median). p was calculated from Kruskal–Wallis test.

Appendix Table 2: Laboratory data of 42 patients with server COVID-19 at baseline

	**Standard of Care plus GC (n=28)**	**Standard of Care (n=14)**
**Blood routine**		
Haemoglobin, g/L	130 (124-146)	127 (119-135)
WBC count, $\times10^9$/L	5.58 (4.73-7.11)	5.49 (4.48-5.97)
Lymphocyte count, $\times10^9$/L	1.04 (0.72-1.29)	1.21 (0.8-1.37)
Neutrophil count, $\times10^9$/L	4.32 (2.8-5.55)	3.6 (2.48-4.73)
Haematocrit, %	38.9 (37.5-43.2)	39.3 (36.9-40.7)
Platelets, $\times10^9$/L	206 (164-262)	186 (181-256)
**Coagulation function**		
Activated partial thromboplastin time, s	30.6 (28.7-34)	30.4 (29-36.6)
Prothrombin time, s	13.4 (12.6-14.1)	12.7 (11.7-13.8)
INR	1.13 (1.05-1.18)	1.06 (0.97-1.15)
**Blood biochemistry**		
Alanine aminotransferase, U/L	25 (14-44)	19 (13.5-34.5)
Total Bilirubin, mmol/L	10.7 (8.7-14.1)	10.7 (8-12.05)
Aspartate aminotransferase, U/L	32 (25-52)	23 (19-29.5)
Glucose, mmol/L	7.25 (5.67-9.49)	5.57 (5.43-5.90)
Blood Urea Nitrogen (urea), mmol/L	5.37 (4.46-6.69)	4.14 (3.36-5.08)
Serum Creatinine, µmol/L	77.10 (60-87.7)	62.35 (57.7-70.55)
Lactate dehydrogenase, U/L	277 (220-393)	219 (189-303)
Creatine kinase, U/L	76 (60-87.7)	74 (32-91)
Sodium, mmol/L	138.8 (137.6-140.3)	139.5 (137.2-142.45)
Potassium, mmol/L	3.62 (3.18-3.85)	3.77 (3.46-3.97)
**Infection-related biomarkers**		
Procalcitonin, ng/mL	0.056 (0.022-0.083)	0.033 (0.02-0.05)
C-reactive protein, mg/L	26.8 (7.63-39.6)	23.33 (3.18-50.77)
HSCRP	47.85 (8.16-89.57)	14.05 (6.78-29.65)
Erythrocyte sedimentation rate, mm/h	45 (14-85)	49 (21-103.6)

INR, International Normalized Ratio; IQR, interquartile range; HSCRP, high-sensitivity C-reactive protein; Data are presented as median (IQR) unless otherwise indicated.

Appendix Table 3: The frequency of complications for the GC plus group vs the standard of care alone group using descriptive statistics for the intention-to-treat populations

Complications no. (%)	Standard of Care plus GC (n=28)	Standard of Care (n=14)	Difference (95%CI)*	P Value
Bacterial pneumonia	17 (60.71)	7 (50.00)	10.71 (-21.12 to 42.55)	0.508
Cardiac arrhythmia	10 (35.71)	6 (42.86)	-7.14 (-30.56 to 24.27)	0.653
Liver dysfunction	7 (25.00)	2 (14.29)	10.71 (-13.64 to 35.07)	0.692
Bacteremia	5 (17.86)	1 (7.14)	10.71 (-8.86 to 30.29)	0.645
Bronchiolitis	5 (17.86)	1 (7.14)	10.71 (-8.86 to 30.29)	0.645
Coagulation disorder / Disseminated Intravascular Coagulation	1 (3.57)	2 (14.29)	-10.71 (-30.29 to 8.86)	0.254
Hyperglycemia	2 (7.14)	0	7.14 (-2.40 to 16.68)	0.545
Myocardial ischemia	0	1 (7.14)	-7.14 (-20.63 to 6.35)	0.333

* Calculated using a generalized linear model.

Appendix Table 4: The frequency of treatments for the GC plus group vs the standard of care alone group in the intention-to-treat populations

Treatment no. (%)	Standard of Care plus GC (n=28)	Standard of Care (n=14)	Difference (95%CI)*	*P* value
Antiviral agents	27 (96.43)	12 (85.71)	10.71 (-8.86 to 30.29)	0.2037
Oxygen therapy	26 (92.86)	12 (85.71)	7.14 (-13.52 to 27.81)	0.4572
Antibiotics	23 (82.14)	10 (71.43)	10.71 (-16.88 to 38.30)	0.4250
Corticosteroids	10 (35.71)	3 (21.43)	14.29 (-13.59 to 42.16)	0.3451
Non-invasive ventilation	2 (7.14)	1 (7.14)	0 (-16.52 to 16.52)	>0.999

* Calculated using a generalized linear model.

Appendix Table 5: Laboratory data of 40 patients with server COVID-19 at day 7*

	Standard of Care plus GC (n=28)	Standard of Care (n=14)	Difference (95%CI)**	*P* value
**Blood routine**				
Haemoglobin, g/L	124 (109-134)	123 (111-134)	0 (-13 to 12)	0.9353
WBC count, $\times 10^9$/L	7.66 (5.79-8.56)	5.44 (3.94-8.30)	1.79 (-0.27 to 4.08)	0.1047
Lymphocyte count, $\times 10^9$/L	1.31 (0.88-1.60)	1.27 (1.14-1.77)	-0.07 (-0.53 to 0.27)	0.6969
Neutrophil count, $\times 10^9$/L	5.33 (4.32-6.91)	4.20 (2.45-6.31)	1.38 (-0.56 to 3.33)	0.1679
Haematocrit, %	37.8 (33.3-41.3)	37.55 (33.80-41.60)	0.10 (-4.10 to 3.90)	0.9741
Platelets,$\times 10^9$/L	281 (221-343)	288 (213.5-339.5)	8.50 (-68 to 92)	0.8711
**Coagulation function**				
Activated partial thromboplastin time, s	30.3 (29.3-31.4)	32.10 (29.45-34.55)	-1.55 (-4.50 to 1.20)	0.2812
Prothrombin time, s	12.4 (11.6-13.0)	12.45 (12-13.6)	-0.25 (-1.20 to 0.70)	0.5776
INR	1.04 (0.96-1.09)	1.04 (1.0-1.14)	-0.02 (-0.10 to 0.06)	0.5894
**Blood biochemistry**				
Alanine aminotransferase, U/L	28 (16-49)	21 (12-36.5)	7 (-6.0 to 22.0)	0.2204
Total Bilirubin, mmol/L	13.35 (8.45-16.9)	15.15 (8.9-18.55)	-0.5 (-5.6 to 3.9)	0.7498
Aspartate aminotransferase, U/L	22 (17-29.5)	22 (17-32)	0 (-7.0 to 6.0)	0.9732
Glucose, mmol/L*	43.83 (43.83-43.83)	5.57 (5.43-5.90)		
Blood Urea Nitrogen (urea), mmol/L	5.16 (3.82-6.35)	4.14 (3.36-5.08)	1.09 (-0.28 to 2.49)	0.1309
Serum Creatinine, µmol/L	69.20 (57.95-76.7)	62.35 (57.7-70.55)	6.25 (-3.1 to 18.3)	0.1604
Lactate dehydrogenase, U/L	218.5 (188-600)	219 (189-303)	23.5 (-13 to 985)	>0.999
Creatine kinase, U/L	38.5 (36-44)	74 (32-91)	-4.0 (-17.0 to 862)	0.3559
Sodium, mmol/L	139.9 (138.5-140.8)	139.5 (137.2-142.45)	0 (-1.6 to 1.5)	0.9482
Potassium, mmol/L	4.05 (3.9-4.3)	3.77 (3.46-3.97)	0.17 (-0.11 to 0.45)	0.2175
**Infection-related biomarkers**				
Procalcitonin, ng/mL	0.02 (0.02-0.05)	0.012 (0.02-0.03)	0 (0 to 0.03)	0.3229
C-reactive protein, mg/L	3.76 (2.68-12.39)	6.35 (3.02-19.83)	-1.63 (-6.62 to 1.30)	0.3295
HSCRP	3.11 (3.11-9.83)	3.30 (3.30-3.30)	-0.19 (-0.19 to 146.7)	0.7557
Erythrocyte sedimentation rate	28.55 (18.25-56.05)	41.8 (41-51.50)	-12.45 (-29.3 to 13.2)	0.3081

INR, International Normalized Ratio; IQR, interquartile range; HSCRP, high-sensitivity C-reactive protein; Data are presented as median (IQR) unless otherwise indicated. Two patients died during 7-day treatment (Standard of Care plus GC, n=1; Standard of Care only, n=1).

**Hodges–Lehmann estimate of location shift (pseudo-median). p was calculated from Kruskal–Wallis test.

*one case in Standard of Care plus GC group;
