# Supplementary material for: Guideline-Based Chinese Herbal Medicine Treatment Plus Standard Care for Severe Coronavirus Disease 2019 (G-CHAMPS): Evidence From China
Source: Front Med (Lausanne). 2020 May 27;7:256. doi: 10.3389/fmed.2020.00256 (PMC7267028; doi:10.3389/fmed.2020.00256)
Supplement: Supplementary file 3 [file Data_Sheet_3.PDF]

Supplemental Appendix

Members of the G-CHAMPS Collaborative Group

The G-CHAMPS trial was conducted by Dongzhimen Hospital, Beijing University of Chinese Medicine and implemented in close partnership with Hubei Provincial of Integrated Chinese and Western Medicine, China. In addition to the core writing group, the following study group members also contributed substantively to the conduct of the G-CHAMPS trial. These authors contributed equally to this work. All the authors dedicated large amounts of time to the study, in the hope of improving care for patients during COVID-19 outbreak. All members read and approved the final report. All authors agree, as the G-CHAMPS group members, to submit this article.

These authors contributed equally to this work, and are presented in alphabetical order.

Name	Name (Chinese characters)	Degree	Affiliation	Email
An, Changqing	安长青	Ph.D	Hubei Provincial Hospital of Integrated Chinese and Western Medicine	2642037775@qq.com
Chen, Ying	陈莹	M.D.	Dongzhimen Hospital, Beijing University of Chinese Medicine	chenying0206@126.com
Dai, Hengheng	代恒恒	M.D.	Dongzhimen Hospital, Beijing University of Chinese Medicine	csdy1114@sina.com
Du, Jian	杜渐	M.D.	Dongzhimen Hospital, Beijing University of Chinese Medicine	dujian8412@126.com
Geng, Xu	耿旭	B.S.	Dongzhimen Hospital, Beijing University of Chinese Medicine	470601647@qq.com
Hao, Xuezeng	郝学增	M.D.	Dongzhimen Hospital, Beijing University of Chinese Medicine	haoxuezeng@163.com
He, Weili	赫伟丽	M.D.	Dongzhimen Hospital, Beijing University of Chinese Medicine	heweili2004@163.com
Ji, Chunyan	计春燕	M.D.	Hubei Provincial Hospital of Integrated Chinese and Western Medicine	574900945@qq.com
Ji, Dongmei	纪冬梅	B.S.	Dongzhimen Hospital, Beijing University of Chinese Medicine	460600663@qq.com
Liang, Tengxiao	梁腾霄	Ph.D	Dongzhimen Hospital, Beijing University of Chinese Medicine	13601133923@163.com

Name	Name (Chinese characters)	Degree	Affiliation	Email
Liu, Yan	刘岩	M.D.	Dongzhimen Hospital, Beijing University of Chinese Medicine	sasliu@yeah.net
Shang, Hongcai	商洪才	Ph.D	Dongzhimen Hospital, Beijing University of Chinese Medicine	shanghongcai@126.com
Tian, Guihua	田贵华	Ph.D	Dongzhimen Hospital, Beijing University of Chinese Medicine	rosetgh@163.com
Wang, Tong	王彤	B.S.	Dongfang Hospital, Beijing University of Chinese Medicine	wdwt388@sina.com
Wang, Xian	王显	Ph.D	Dongzhimen Hospital, Beijing University of Chinese Medicine	wx650515@163.com
Wu, Huayang	吴华阳	Ph.D	Dongzhimen Hospital, Beijing University of Chinese Medicine	wuhuayang2003095@126.com
Xia, Wenguang	夏文广	Ph.D	Hubei Provincial Hospital of Integrated Chinese and Western Medicine	docxwg@163.com
Ye, Yongan	叶永安	Ph.D	Dongzhimen Hospital, Beijing University of Chinese Medicine	Dzmscholar@163.com
Zhang, Chi	张弛	Ph.D	Dongzhimen Hospital, Beijing University of Chinese Medicine	saga618@126.com
Zhang, Jingjing	张晶晶	M.D.	Dongzhimen Hospital, Beijing University of Chinese Medicine	zjhzyyfy@163.com
Zhang, Xiaoyu	张晓雨	Ph.D	Dongzhimen Hospital, Beijing University of Chinese Medicine	zbzhangxiaoyu@aliyun.com
Zhang, Xuecheng	张学成	M.D.	Dongzhimen Hospital, Beijing University of Chinese Medicine	zhangxuechengmail@163.com
Zhang, Yaosheng	张耀圣	Ph.D	Dongzhimen Hospital, Beijing University of Chinese Medicine	ysz3129@163.com
Zhang, Ziwei	张子伟	B.S.	Dongzhimen Hospital, Beijing University of Chinese Medicine	15910802923@163.com
Zhou, Kehua	周科华	MD, DPT	Catholic Health System Internal Medicine Training Program, University at Buffalo, Buffalo, NY, USA	kehuazho@buffalo.edu

Manuscript title: Guideline-based Chinese Herbal Medicine treatment Plus Standard care for severe coronavirus disease 2019 (G-CHAMPS)

Corresponding author: Yong-an Ye, PhD, Dongzhimen Hospital, Beijing University of Chinese Medicine.

Name (姓名汉字)	Name (姓名拼音)	Degree (最高学历)	Affiliation (工作单位)	Email (邮箱)
[illegible]	Shang Hongcai	Ph.D	Dongzhimen Hospital, Beijing University of Chinese Medicine	shanghongcai@126.com
刘岩	Liu Yan	M.D	Dongzhimen Hospital, Beijing University of Chinese Medicine	sasliu@yeah.net
周科华	Kehua Zhou	MD, DPT	Catholic Health System Internal Medicine Training Program, University at Buffalo, Buffalo, NY, USA	kehuazho@buffalo.edu
张弛	Zhang Chi	Ph.D	Dongzhimen Hospital, Beijing University of Chinese Medicine	saga618@126.com
张晓雨	Zhang Xiaoyu.	Ph.D	Dongzhimen Hospital, Beijing University of Chinese Medicine	zbzhangxiaoyu@aliyun.com
梁腾霄	Liang Tengxiao	Ph.D	Dongzhimen Hospital, Beijing University of Chinese Medicine	13601133923@163.com

Name (姓名汉字)	Name (姓名拼音)	Degree (最高学历)	Affiliation (工作单位)	Email (邮箱)
郝学增	Xue-zeng, Hao	M.D.	Dongzhimen Hospital, Beijing University of Chinese Medicine	haoxuezeng@163.com
纪冬梅	Ji dongmei	B.S.	Dongzhimen Hospital, Beijing University of Chinese Medicine	460600663@qq.com
耿旭	Geng Xu	B.S.	Dongzhimen Hospital, Beijing University of Chinese Medicine	470601647@qq.com
杜健	Du Jian	M.D.	Dongzhimen Hospital, Beijing University of Chinese Medicine	dujian8412@126.com
张子祎	Zhang zi wei	B.S.	Dongzhimen Hospital, Beijing University of Chinese Medicine	15910802923@163.com
王彤	Wang Tong	B.S.	Dongfang Hospital, Beijing University of Chinese Medicine	wdwt388@sina.com
安长青	An changqing	Ph.D	Hubei Provincial Hospital of Integrated Chinese and Western Medicine	2642037775@qq.com
夏文广	XIA WENGUANG	Ph.D	Hubei Provincial Hospital of Integrated Chinese and Western Medicine	docxwg@163.com
计春燕	Ji Chunyan	M.D.	Hubei Provincial Hospital of Integrated Chinese and Western Medicine	574900945@qq.com

Name (姓名汉字)	Name (姓名拼音)	Degree (最高学历)	Affiliation (工作单位)	Email (邮箱)
张耀圣	zhang yao sheng	Ph.D	Dongzhimen Hospital, Beijing University of Chinese Medicine	ysz3129@163.com
王显	Wang xian	Ph.D	Dongzhimen Hospital, Beijing University of Chinese Medicine	wx650515@163.com
叶永安	ye yongan	Ph.D	Dongzhimen Hospital, Beijing University of Chinese Medicine	dzmscholar@163.com
陈莹	CHEN YING	M.D.	Dongzhimen Hospital, Beijing University of Chinese Medicine	chenying0206@126.com
张晶晶	ZHANG JINGJING	M.D.	Dongzhimen Hospital, Beijing University of Chinese Medicine	zjhzyyfy@163.com
代恒恒	DAI HENGHENG	M.D.	Dongzhimen Hospital, Beijing University of Chinese Medicine	csdy1114@sina.com
张学成	ZHANG XUECHENG	M.D.	Dongzhimen Hospital, Beijing University of Chinese Medicine	zhangxuechengmail@163.com
田贵华	Tian GuiHua	Ph.D	Dongzhimen Hospital, Beijing University of Chinese Medicine	rosetgh@163.com
