# Supplementary figures and images for: Guideline-Based Chinese Herbal Medicine Treatment Plus Standard Care for Severe Coronavirus Disease 2019 (G-CHAMPS): Evidence From China
Source: Front Med (Lausanne). 2020 May 27;7:256. doi: 10.3389/fmed.2020.00256 (PMC7267028; doi:10.3389/fmed.2020.00256)

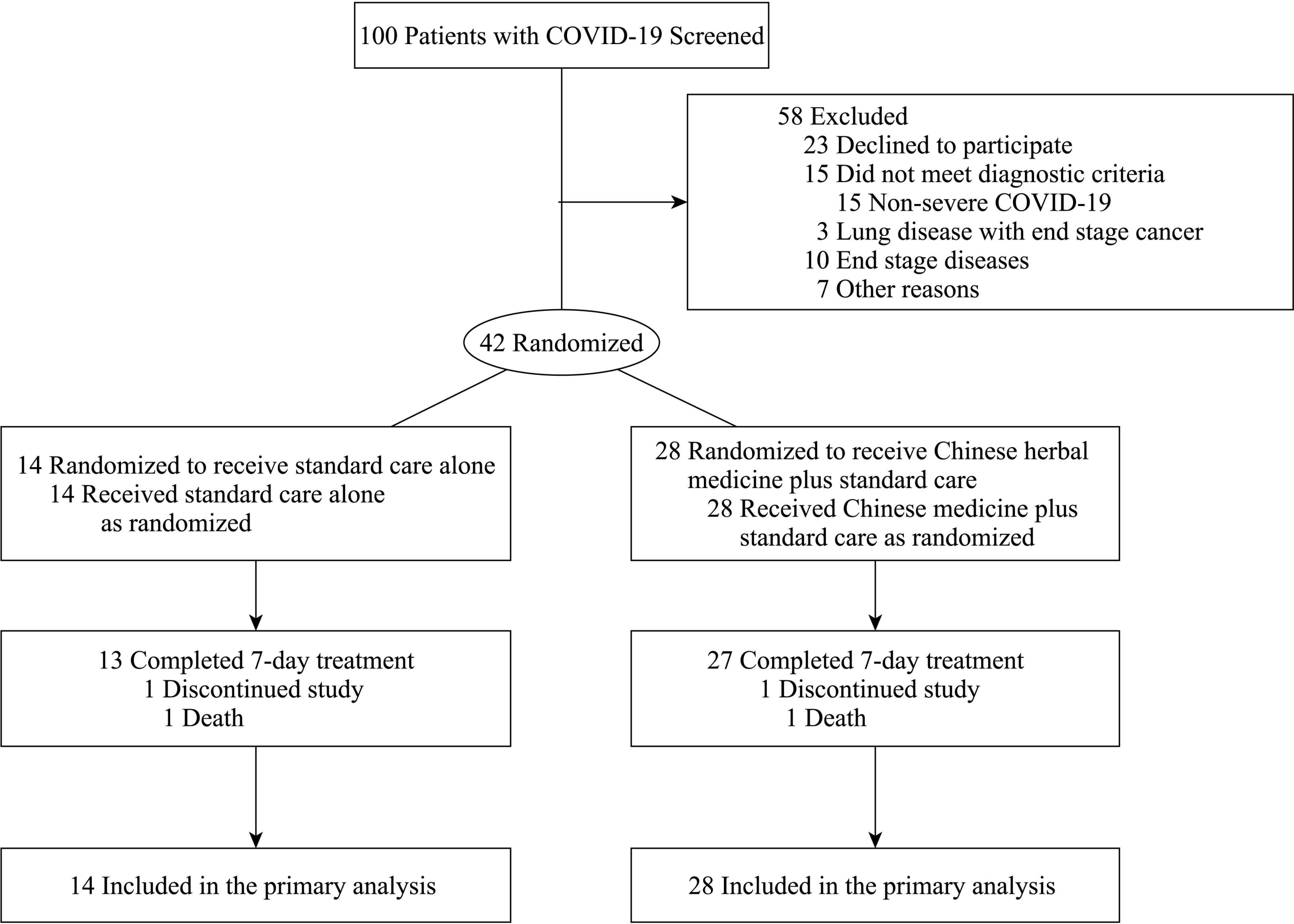

Supplement: Supplementary file 4 [file Data_Sheet_4.PDF]
